# Need equality and access equity to assistive products across genders and locations in 29 countries: a cross-sectional study

**DOI:** 10.3389/fpubh.2025.1581048

**Published:** 2025-08-08

**Authors:** Johan Borg, Sébastien Muller, Arne H. Eide, Luc de Witte, Irene Calvo, Chapal Khasnabis, Wei Zhang

**Affiliations:** ^1^School of Health and Welfare, Dalarna University, Falun, Sweden; ^2^SINTEF Digital, Trondheim, Norway; ^3^SINTEF Digital, Oslo, Norway; ^4^The Hague University of Applied Sciences, The Hague, Netherlands; ^5^Access to Assistive Technology, World Health Organization (WHO), Geneva, Switzerland

**Keywords:** access, assistive products, assistive technology, equality, equity, gender, need, location

## Abstract

**Background:**

Evidence from nationally representative assistive product studies on needs equality and access equity is essential for effectively targeting measures by health and other services to improve access to assistive products. This multi-country study explored equality regarding the need for and equity regarding access to assistive products across genders and locations.

**Materials and methods:**

This cross-sectional study analyzed aggregated self-reported data from 24 nationally and five sub-nationally representative surveys in countries with Human Development Index (HDI) ranging from 0.452 to 0.945. In 27 surveys, participants of all ages had been recruited through two-stage cluster sampling, while in two surveys participants aged 18 and above were recruited through simple random sampling. Individual-level data were collected through the Rapid Assistive Technology Assessment (rATA) questionnaire using personal, telephone, or web interviewing in 2019–2021. The main outcomes were assistive product needs inequality and access inequity, defined as the ratio of the difference in need or access between two sub-populations to the need or access in the total population.

**Results:**

Data were collected from 323,647 individuals of whom 44.9%−57.2% were women and 10.1%−89.5% lived in rural locations across the countries. Although varying considerably between countries, the need for assistive products including spectacles was generally higher among women while access was lower in rural areas and among women. Excluding spectacles, the need was higher and the access was lower in rural areas and among women. The access inequity between rural and urban areas was large (26% of the median access including spectacles, and 42% excluding spectacles) while it was smaller between women and men (6.4% including and 13% excluding spectacles). Access inequity decreased with increasing HDI.

**Conclusion:**

In efforts to achieve universal access to assistive products, especially location but also gender ought to be considered.

## 1 Introduction

Worldwide, an estimated 2.5 billion people need assistive products comprising any external products with a primary purpose of maintaining or improving functioning and independence, thereby promoting wellbeing (1). Assistive products can also be used to prevent impairments and secondary health conditions (1). They constitute a means for exercising human rights, which is why the UN Convention on the Rights of Persons with Disabilities (UNCRPD) requires states to provide assistive products (2, 3). They are instrumental in achieving all sustainable development goals, emphasizing the importance of universal access to assistive products to ensure no one is left behind (4).

As part of universal health coverage, universal access to assistive products is a state where everyone, everywhere receives the assistive products they need without financial or other hardships (5). To monitor this, data on access to assistive products are required. However, population data on levels of access have been scarce, and where collected, different methods have been used (1, 6–8). Comparing country situations has therefore been problematic.

To address the lack of comparable data, the World Health Organization (WHO) launched a multi-country study in 2021, supporting countries and other actors in collecting self-reported population data on access to assistive products using the Rapid Assistive Technology Assessment (rATA) questionnaire (1, 9, 10). The study found that the need for and access to assistive products varied considerably across the 29 surveyed countries. In about half of these countries, less than a quarter of the people expressing a need for assistive products had access to the assistive products they needed, and in one of the countries, only 3% had such access. Access increased with higher levels of development as measured by the Human Development Index (HDI). Besides differences between countries, certain differences in needs and access were observed between women and men, and between rural and urban populations (1). However, the extent to which these differences constitute inequality in needs or inequity in access was not analyzed in detail.

Knowledge about the needs for assistive products and their distribution in a population, as well as disparities in access to assistive products, is critical to planning and implementing effective strategies for improving access to assistive technology, which includes assistive products and services and systems for their provision (1). Such evidence is a prerequisite to support the realization of the World Health Assembly resolution WHA 71.8, which calls for countries to improve access to assistive technology by developing, implementing, and strengthening policies and programs within universal health or social services coverage (11). To contribute to building the required evidence, the objective of this study was to explore equality regarding the need for and equity regarding access to assistive products across genders and locations in countries where representative rATA surveys have been undertaken.

## 2 Materials and methods

### 2.1 Study design and setting

This cross-sectional study analyzed aggregate data collected through nationally representative surveys in 24 countries and regionally representative surveys in five countries conducted in 2019–2021. Located in all six WHO regions, the countries varied in population size and development level. The surveys were carried out to inform the development of the WHO and UNICEF Global Report on Assistive Technology (1).

### 2.2 Participants

In 27 countries, the sample frame included all people, and in two countries, the sample frame included people aged 18 and above. In one of the last two countries, the sample included people having a mobile phone subscription only.

### 2.3 Procedures

The method for collecting data on the need for and access to assistive products using the rATA tool (10) is described in (1) and (9). Computer-assisted personal interviewing (CAPI) was used in 24 countries, and paper-based personal interviews were conducted in one country. In these countries, all household members were interviewed. Computer-assisted telephone interviewing (CATI) of one person per household was used in two countries. CATI in combination with CAPI was used in one country, and computer-assisted web interviewing (CAWI) in combination with CATI was used in one country. Not all members of a household were interviewed in the last two countries.

### 2.4 Ethics

A general ethical approval was obtained for the surveys from the WHO Ethics Review Committee (protocol ID ATMrATA approved on 23 June 2021; protocol ID rATA2Ana on 8 December 2022), and individual ethical approvals were obtained from concerned authorities in each surveyed country. This study used public country-level data available at the WHO Global Health Observatory.

### 2.5 Outcomes

As the need for spectacles was large compared to other assistive products, analysis of aggregate data both with and without spectacles was undertaken. The high need for and relatively greater access to spectacles could otherwise skew the results for other assistive products. The need for and access to assistive products with and without spectacles were defined as follows (1):

*Need including spectacles* = Proportion of a population using or reporting a need for at least one assistive product, which may include spectacles. Range: 0% to 100%
*Need excluding spectacles* = Proportion of a population using or reporting a need for at least one assistive product other than spectacles. Range: 0% to 100%
*Access including spectacles* = Ratio of the proportion of a population using assistive products, which may include spectacles, that do not report a need for new or additional assistive products to the proportion of a population using or reporting a need for at least one assistive product, which may include spectacles. Range: 0% to 100%
*Access excluding spectacles* = Ratio of the proportion of a population using assistive products other than spectacles that do not report a need for new or additional assistive products to the proportion of a population using or reporting a need for at least one assistive product other than spectacles. Range: 0% to 100%

To measure inequality and inequity, this study introduces *need inequality* and *access inequity* as the main outcomes:

*Need inequality* reflects how the need for assistive products differs between two subpopulations. It represents the difference in need between two subpopulations expressed as a fraction of the need in the total population.
*Access inequity* reflects how access to assistive products differs between two subpopulations. It represents the difference in access between two subpopulations expressed as a fraction of the access in the total population.

If the need is equal or access is equitable between two subpopulations, the value of *need inequality* or *access inequity* equals 0. A positive or negative value indicates that the need or access is higher in one of the two compared subpopulations. Compared to using absolute differences between subpopulations, fractions more clearly reveal small differences between the subpopulations when the values of need and access in the total population are low.

*Need inequality* and *access inequity*, with and without spectacles, for comparisons between rural and urban populations and between female and male populations, are defined as follows:

*N*_*r*−*u, i*_ = (Need including spectacles among the rural population – Need including spectacles among the urban population)/(Need including spectacles among the total population)
*N*_*r*−*u, e*_ = (Need excluding spectacles among the rural population – Need excluding spectacles among the urban population)/(Need excluding spectacles among the total population)
*N*_*f*−*m, i*_ = (Need including spectacles among the female population – Need including spectacles among the male population)/(Need including spectacles among the total population)
*N*_*f*−*m, e*_ = (Need excluding spectacles among the female population – Need excluding spectacles among the male population)/(Need excluding spectacles among the total population)
*A*_*r*−*u, i*_ = (Access including spectacles among the rural population – Access including spectacles among the urban population)/(Access including spectacles among the total population)
*A*_*r*−*u, e*_ = (Access excluding spectacles among the rural population – Access excluding spectacles among the urban population)/(Access excluding spectacles among the total population)
*A*_*f*−*m, i*_ = (Access including spectacles among the female population – Access including spectacles among the male population)/(Access including spectacles among the total population)
*A*_*f*−*m, e*_ = (Access excluding spectacles among the female population – Access excluding spectacles among the male population)/(Access excluding spectacles among the total population)

Given that the access inequity equals zero (*A* = 0) when the access is equal for two population groups, and that there is at least some inequity if access inequity is negative or positive (*A* ≠ 0), it is useful to analyze the absolute value of *A* (|*A*|). The absolute access inequity increases with increasing access gaps between two subpopulations, irrespective of which subpopulation has a higher level of access.

To explore how *need inequality* and *access inequity* correlate with a country's level of development, this study used *HDI* as an independent variable as it has been found to correlate with a country's level of access (1). Ranging from 0 to 1, *HDI* is the geometric mean of normalized indices of the three dimensions: long and healthy life (life expectancy at birth), knowledge (expected years of schooling; mean years of schooling), and a decent standard of living (gross national income per capita) (12).

### 2.6 Statistical analysis

The sample sizes varied between 1,479 and 62,723 among the 29 included countries. As a general recommendation for rATA household surveys using two-stage cluster sampling, the sample size in each country should be 13,392 (9). This was based on a conservative assumption that 1% of the global population had access to the assistive products they need, and an ambition to measure the level of access with 95% confidence with a precision of 25%. Assuming that the conservative sample design effect (*f* = 2) remained and given that the measured access in the 29 rATA surveys ranged from 2.6% to 89.8% including spectacles, and 2.1% to 83.5% excluding spectacles, all surveys achieved the desired precision for access including spectacles, and all surveys except one (Liberia) achieved it for access excluding spectacles (1).

Secondary analyses were performed on the data from the 29 countries using IBM SPSS Statistics version 28.0.1.0 (13). As 5 of the 8 need and access variables were not normally distributed (skewness below −2 or kurtosis above 2), nonparametric tests were used. The analyses included descriptive statistics, one-sample Wilcoxon signed rank tests with a hypothesized median of 0, and Spearman's rank correlation tests. A test was considered statistically significant if the *p*-value was equal to or below 0.05. All data were weighted at the country level to represent each population more accurately.

## 3 Results

**Figure 1:**
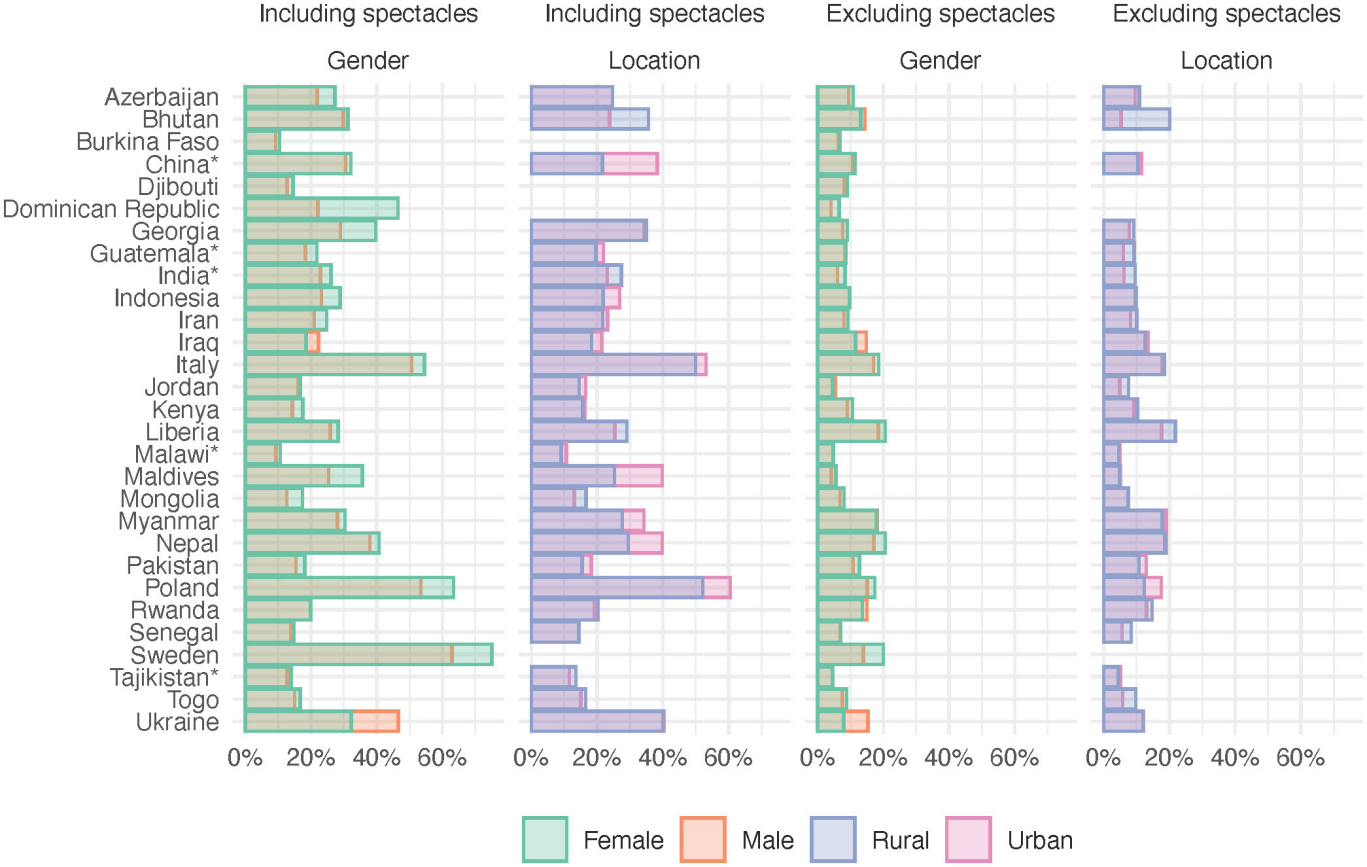
Need including and excluding spectacles by country disaggregated by gender and location. ^*^Provincial surveys, see Table 1.

**Figure 2:**
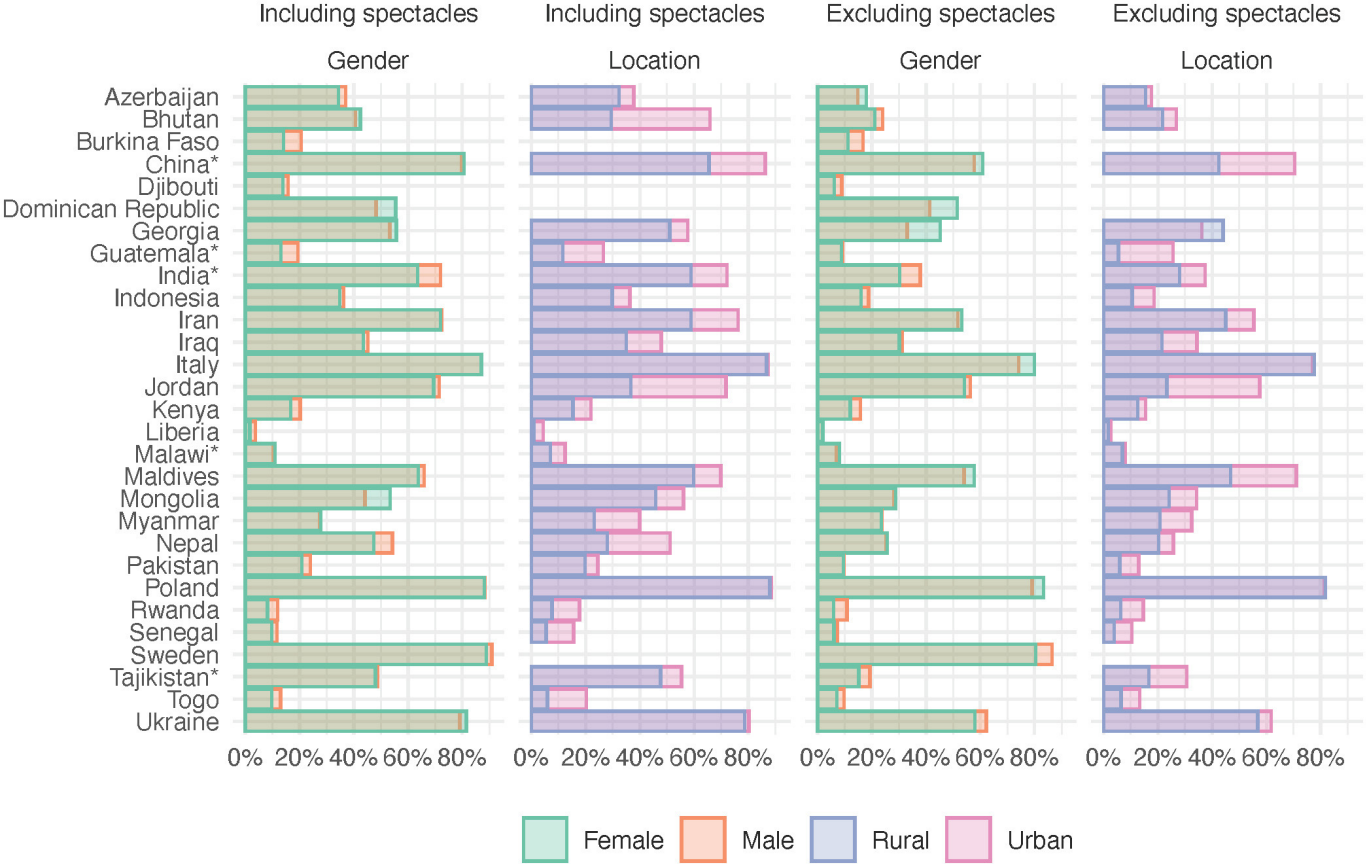
Access including and excluding spectacles by country disaggregated by gender and location. ^*^Provincial surveys, see Table 1.

The surveyed countries, their *HDI*s, and the number of participants are presented in Table 1 along with the proportions of rural and female participants and *need inequality* and *access inequity* values. Data on location were missing from four countries. Levels of need for and access to assistive products are shown in Figures 1, 2. In countries like Italy and Poland, all *access inequity* values were close to zero, indicating rather equitable access to assistive products. In almost all countries, location-related *access inequity* deviated more from 0% than the gender-related values, indicating that location-related inequity was generally larger than gender-related inequity. For example, in Myanmar, location-related inequity was 35–40 times higher than gender-related inequity (60.4% vs. 1.5% including spectacles, and 49.6% vs. 1.4% excluding spectacles).

**Table 1:** Country characteristics, need inequality, and access inequity.

**Spectacles**					**Need inequality**				**Access inequity**			
					**Rural vs. Urban**		**Female vs. Male**		**Rural vs. Urban**		**Female vs. Male**	
					**Incl**.	**Excl**.	**Incl**.	**Excl**.	**Incl**.	**Excl**.	**Incl**.	**Excl**.
**Country (provinces)**	**HDI**	**Participants**	**Rural (%)**	**Female** **(%)**	***N**_*r*−*u, i*_ **(%)***	***N**_*r*−*u, e*_* ***(%)***	***N**_*f*−*m, i*_ **(%)***	***N**_*f*−*m, e*_* ***(%)***	***A**_*r*−*u, i*_ **(%)***	***A**_*r*−*u, e*_* ***(%)***	***A**_*f*−*m, i*_ **(%)***	***A**_*f*−*m, e*_* ***(%)***
Azerbaijan	0.756	5,586	45.5	51.1	−0.1	12.1	21.7	13.8	−15.2	−13.2	−7.4	18.5
Bhutan	0.654	11,930	58.5	52.4	38.8	107.7	4.4	−9.8	−87.3	−22.5	4.4	−12.8
Burkina Faso	0.452	15,043	–	52.0	–	–	12.0	10.2	–	–	−37.7	−40.5
China (Anhui, Beijing, Fujian, Hubei, Sichuan, Shaanxi)	0.778	15,057	41.8	50.4	−53.4	−9.6	5.0	5.6	−25.8	−47.0	1.1	5.3
Djibouti	0.524	11,720	–	51.9	–	–	12.6	10.2	–	–	−12.6	−36.7
Dominican Republic^1^	0.756	5,003	–	52.4	–	–	69.8	44.8	–	–	13.7	20.9
Georgia	0.812	6,864	42.8	56.4	01.9	16.2	31.3	16.7	−12.2	19.7	4.5	30.3
Guatemala (Solola)	0.630	2,868	74.9	55.0	−11.2	39.6	16.9	4.1	−95.4	−221.8	−40.1	−5.8
India (Delhi, Haryana, Gujarat, Odisha, Karnataka)	0.674	8,573	51.3	51.7	0.6	29.6	15.2	31.8	−17.7	−17.8	−13.5	−22.1
Indonesia	0.718	11,300	27.3	53.3	−19.3	−1.5	21.8	−1.4	−18.2	−46.3	−4.2	−15.5
Iran	0.783	18,870	36.0	50.4	−6.5	22.4	16.9	13.7	−24.1	−19.8	−0.6	2.7
Iraq	0.674	14,220	30.3	48.1	−15.1	−7.4	−18.4	−24.5	−29.1	−41.9	−3.8	−3.5
Italy	0.892	10,170	15.6	52.1	−6.1	4.2	7.2	8.5	−0.5	0.7	0.0	7.5
Jordan	0.729	13,416	10.1	50.2	−12.0	53.2	3.4	−17.8	−49.8	−61.8	−2.9	−4.0
Kenya	0.601	12,253	55.3	49.8	−3.7	11.0	20.0	15.9	−36.6	−20.4	−18.5	−27.5
Liberia	0.480	5,207	44.7	54.2	13.7	21.5	8.7	10.2	−129.3	−43.4	−80.2	−5.0
Malawi (Blantyre)	0.541	9,340	37.1	52.3	−15.0	−7.7	12.4	−0.4	−52.1	−13.0	6.4	14.7
Maldives	0.740	6,843	71.1	51.8	−47.1	−8.3	33.6	29.5	−15.7	−42.5	−3.3	6.6
Mongolia	0.737	10,739	56.0	51.4	24.0	−1.0	31.4	16.2	−20.5	−35.8	18.6	2.5
Myanmar	0.583	8,743	76.4	52.7	−22.5	−6.9	7.8	−1.9	−60.4	−49.6	1.5	−1.4
Nepal	0.602	11,230	55.3	52.6	2.4	27.0	4.9	2.1	−35.6	−13.5	−18.6	−20.1
Pakistan	0.557	62,723	51.1	50.4	−16.4	−17.8	15.8	16.1	−21.3	−72.2	−13.8	−3.3
Poland	0.880	6,694	23.9	50.9	−14.3	−32.0	16.9	14.3	−0.6	0.5	−0.3	5.3
Rwanda	0.543	7,156	81.1	57.2	5.2	11.7	−0.4	−9.7	−101.7	−101.2	−37.4	−61.0
Senegal	0.512	10,692	61.0	50.7	1.0	40.8	6.7	3.0	−94.8	−98.6	−17.8	−19.1
Sweden^2^	0.945	1,479	–	50.2	–	–	17.6	36.0	–	–	−2.2	−7.2
Tajikistan (Sughd)	0.688	2,500	89.5	51.2	15.0	−14.0	9.4	−2.2	−16.0	−76.6	−1.4	−22.2
Togo	0.515	10,359	67.6	50.3	9.6	47.9	10.2	15.4	−126.7	−82.0	−29.0	−31.7
Ukraine	0.779	7,069	36.0	44.9	−0.9	−1.2	−35.4	−60.8	−1.9	−8.1	3.0	−6.9

^1^Sample frame: 18 years and above. ^2^Sample frame: 18 years and above, and mobile phone subscription.

**Figure 3:**
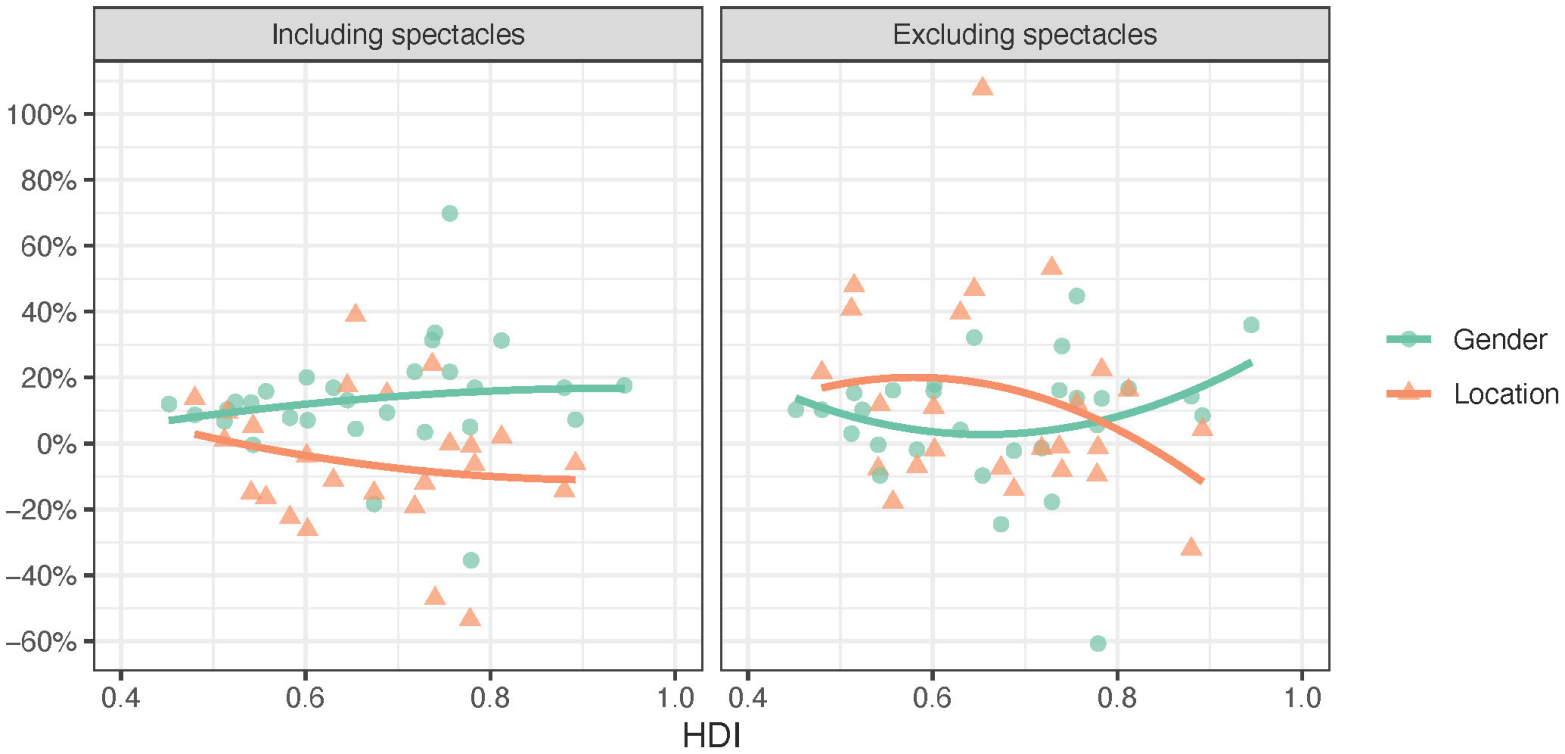
Need inequality including and excluding spectacles by HDI (2^nd^ order trendlines).

Table 2 and Figure 3 reveal that *need inequality* had positive and negative values across the countries, indicating that the rural or female population had a higher need in some countries, and, in others, the urban or male population had a higher need. The medians and the one-sample Wilcoxon signed rank test showed that the need for assistive products excluding spectacles was statistically significantly higher among rural populations in the surveyed countries and that the need for assistive products was statistically significantly higher among women including and excluding spectacles. The correlations between *HDI* and *need inequality* were not statistically significant. However, Figure 3 indicates that rural populations in countries with lower *HDI* tend to have a greater need for assistive products, both including and excluding spectacles, than in countries with higher *HDI*.

**Table 2:** Need inequality and access inequity and their correlation with HDI.

**Spectacles**	**Need inequality**				**Access inequity**			
	**Rural vs. Urban**		**Female vs. Male**		**Rural vs. Urban**		**Female vs. Male**	
	**Incl**.	**Excl**.	**Incl**.	**Excl**.	**Incl**.	**Excl**.	**Incl**.	**Excl**.
**Statistic**	***N**_*r*−*u, i*_*	***N**_*r*−*u, e*_*	***N**_*f*−*m, i*_*	***N**_*f*−*m, e*_*	***A**_*r*−*u, i*_*	***A**_*r*−*u, e*_*	***A**_*f*−*m, i*_*	***A**_*f*−*m, e*_*
Number of countries	25	25	29	29	25	25	29	29
Minimum	−0.534	−0.320	−0.354	−0.608	−1.293	−2.218	−0.802	−0.610
Maximum	0.388	1.077	0.698	0.448	−0.005	0.197	0.186	0.303
Median	−0.037	0.110	0.124	0.102	−0.258	−0.419	−0.033	−0.050
One–Sample Wilcoxon signed rank test, Hypothesized median = 0, Test statistic	−1.359	1.978	3.622	2.368	−4.372	−4.076	−2.476	−1.957
*p* (two–tailed)	0.174	0.048	< 0.001	0.018	< 0.001	< 0.001	0.013	0.050
Spearman's rank correlation test, Coefficient	−0.219	−0.257	0.294	0.227	0.840	0.585	0.612	0.593
*p* (two–tailed)	0.292	0.214	0.122	0.237	< 0.001	0.002	< 0.001	< 0.001

**Figure 4:**
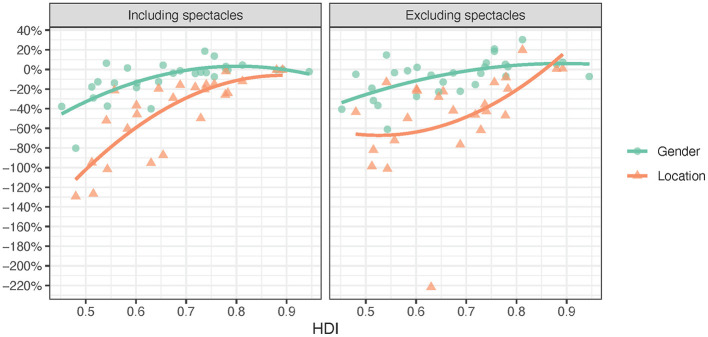
Access inequity including and excluding spectacles by HDI (2^nd^ order trendlines).

Figure 4 and Table 2 show that three of the *access inequity* values were both positive and negative whereas the *access inequity* values for location including spectacles were only negative, i.e., access was higher in urban than rural areas. The access to assistive products was statistically significantly lower for rural populations and women including and excluding spectacles. Median *access inequity* values for location and gender were closer to zero when spectacles were included than when they were excluded. The median *access inequity* values for location (−0.258 including spectacles and −0.419 excluding spectacles) were considerably larger than for gender (−0.033 and −0.050, respectively). *Access inequity* was statistically significantly moderately or strongly associated with *HDI*.

**Figure 5:**
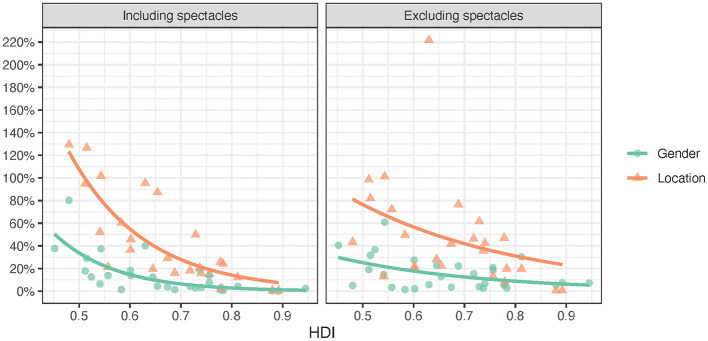
Absolute access inequity including and excluding spectacles by HDI (exponential trendlines).

While Table 1 and Figure 4 present *access inequity* and its direction (positive or negative), Table 3 and Figure 5 present *absolute access inequity* statistics and trends. It shows that median *absolute access inequity* is lowest for gender including spectacles (0.064) and highest for location excluding spectacles (0.419). When excluding spectacles, the median difference in access between rural and urban populations is 41.9% of the average access in the entire population. It can be noted that the absolute access inequity for assistive products including spectacles was 4.0 times higher for location than gender (25.8% vs. 6.4%), and for assistive products excluding spectacles, access inequity was 3.3 times higher for location than gender (41.9% vs. 12.8%).

**Table 3:** Absolute access inequity and its correlation with HDI.

**Spectacles**	**Rural vs. Urban**		**Female vs. Male**	
	**Incl**.	**Excl**.	**Incl**.	**Excl**.
**Statistic**	**|** ***A**_*r*−*u, i*_* **|**	**|** ***A**_*r*−*u, e*_* **|**	**|** ***A**_*f*−*m, i*_* **|**	**|** ***A**_*f*−*m, e*_* **|**
Number of countries	25	25	29	29
Minimum	0.005	0.005	0.000	0.014
Maximum	1.293	2.218	0.802	0.610
Median	0.258	0.419	0.064	0.128
Spearman's rank correlation test, Coefficient	−0.840	−0.549	−0.713	−0.301
*p* (two–tailed)	< 0.001	0.005	< 0.001	0.113

Table 3 shows that three of the *absolute access inequities* were moderately or strongly statistically significantly correlated with *HDI*, whereas the correlation between HDI and access differences between women and men was weak and not statistically significant when spectacles were excluded.

## 4 Discussion

Through measures of need inequality and access inequity, this study explored differences in need for and disparities in access to assistive products using rATA survey data from 29 countries. Including spectacles, the need was higher among women than men, while the access was lower among women and rural populations. No differences in need were found between rural and urban populations. Excluding spectacles, the need was higher and access lower among women and rural populations. The absolute access inequity for assistive products including spectacles was 4.0 times higher for location than gender. For assistive products excluding spectacles, this inequity was 3.3 times higher for location than gender. However, access inequity varied both between and within countries. For example, in one country, the location-related inequity was 35–40 times higher than gender-related inequity. Access inequity correlated with HDI, although the correlation between HDI and absolute access inequity for gender excluding spectacles was not statistically significant. The findings confirm the general observations on differences in need and access between genders and locations as presented in the WHO & UNICEF Global Report on Assistive Technology, which also provides comprehensive recommendations and actions to improve access to assistive products (1).

### 4.1 Discussion of need

The difference in the need for assistive products between women and men may partly be explained by the fact that the need for assistive products is associated with age, and that women live longer than men (1, 14). However, further studies are required to establish the existence and extent of such links. Moreover, it has been suggested that the increased need for assistive products including spectacles in countries with higher HDI may be attributed to a higher prevalence of myopia and near vision impairment, more years in studies, and a higher prevalence of office-based jobs (1).

Though not statistically significant, the association between inequality in the need for assistive products and HDI indicates a tendency in countries with lower HDI to observe a higher need for assistive products in rural populations. In these countries, rural communities often rely on agricultural or labor-intensive jobs, which could increase the risk of physical injuries or disabilities and thereby the need for assistive products (15). This could lead to exacerbated negative consequences from access inequity.

### 4.2 Discussion of access

Although there is a trend of increasing access to assistive products with increasing HDI, it is not the only determinant. Some countries in the low or medium group of the HDI classification achieved access comparable to countries in a higher classification group (1). An analysis of the assistive technology systems in 20 of the included countries found that geographic coverage of assistive technology services is the most instrumental system element and thereby key to equitable access (16). This contributes to explaining access inequity in the disfavor of rural areas found in this study and aligns well with recent literature (7, 17). Moreover, using data from rATA surveys, a range of barriers that impact more negatively on access to assistive products in rural areas compared to urban areas have been identified (18). These barriers include poor availability, high costs, and lack of transportation. Improving access to assistive products and reducing access inequity between rural and urban areas requires an appropriate strategy to address institutional and systemic barriers experienced by rural populations (19). Moreover, fairness of access to assistive products is crucial for the equitable attainment of the sustainable development goals, which can be achieved when the sector develops a stronger systems thinking and market-shaping perspective (20).

The inequity in access to assistive products between rural and urban areas in countries with lower HDI underscores the impact of socioeconomic development on healthcare disparities. Limited economic resources can result in reduced healthcare infrastructure and funding, particularly in rural areas (21). This scarcity of resources can lead to an inadequate supply of assistive products. On average, rural communities tend to have lower income and educational attainment than their urban counterparts (22). Limited financial means may preclude people in rural areas from purchasing assistive products, while lower education levels can hinder their awareness of available options. Furthermore, the social and political context can play a significant role. In countries where urban development is prioritized over rural areas, and where healthcare policies and support are limited, such imbalances can perpetuate disparities. This dynamic is exacerbated by challenges in transportation and infrastructure in rural settings, making it logistically difficult to access healthcare facilities or obtain assistive products. Moreover, gender and ethnic disparities may add layers of disadvantage to rural populations (23).

Recent studies in countries such as Australia, Canada, and England underscore that access to assistive technology is often inequitable and that efforts to achieve equity are required (24–26). To address the inequity in access to assistive products, it is essential to recognize that interventions and policies to reduce inequities in access to healthcare must not limit themselves to intermediary determinants, but must tackle the social mechanisms that systematically produce an inequitable distribution of the determinants of health among population groups (27). Measures of access inequity can be used to guide country-specific policies and strategies to achieve equity in assistive product access.

Previous studies have identified barriers to accessing assistive products, such as the products themselves, challenges in procurement and delivery, capacity gaps in the workforce, failed markets, governance and funding issues, and sociodemographic barriers (1). Reducing location-based access inequity by improving geographic coverage requires appropriate policies, sufficient funding, adequately trained personnel, and the availability of assistive products in need (16). However, there is a need to understand the mechanisms of barriers and their consequences for access to assistive products for different subpopulations. Developing such knowledge is crucial to devising global and national strategies and utilizing available resources to effectively improve equitable access to assistive products.

### 4.3 Limitations and strengths

This study was based on a unique set of representative surveys from 29 countries. As measures of imprecision were not available, it was not possible to provide information on precision for need inequality and access inequity. However, with an exception for assistive products excluding spectacles in one country, the required sample sizes to reach the desired precision to determine population needs for assistive products were achieved.

The limitations of the rATA surveys have been reported in previous studies (1, 9). They include possible inconsistencies in the translation of the rATA questionnaire. However, using a common definition of the need for and use of assistive products, the rATA questionnaire overcomes a limitation that has been seen in previous studies, namely the variation in the definition of indicators of access to assistive products, which have likely led to over- and underestimates (28).

The countries included in this study exhibit a diverse range of health, education, and socioeconomic statuses, as measured by the HDI, ranging from 0.480 to 0.945. As a result, the findings, particularly the correlations as well as the absence of correlations between HDI on the one hand and access inequity and need inequality, respectively, on the other, are relevant and provide important insights at a global level.

### 4.4 Conclusion

Analyses of data from 29 countries found that needs for and access to assistive products vary between countries, genders, and locations. Notably, the disparity in access was significantly more pronounced between different locations than between genders, with the gap being three to four times larger. A country's level of development is positively correlated with access equity, although variations exist both within and across countries. Recognizing these nuances, it is imperative to tailor policies, strategies, and their implementation to the specific conditions of each context, including the economic capacity, when addressing access inequities at the global or national level. Achieving universal access to assistive products requires approaches that address geographical differences while also considering gender.

## Data Availability

The rATA data are openly available at the Assistive technology data portal of the WHO Global Health Observatory (https://www.who.int/data/gho/data/themes/assistivetech).

## References

[1] WHO. Global report on assistive technology. Geneva: World Health Organization (2022).

[2] UN. Convention on the rights of persons with disabilities. New York: United Nations (2006). 10.1515/9783110208856.203 18348362

[3] Borg J Larsson S Östergren PO. The right to assistive technology: for whom, for what, and by whom? Disabil Soc. (2011) 26:151–67. 10.1080/09687599.2011.543862

[4] Tebbutt E Brodmann R Borg J MacLachlan M Khasnabis C Horvath R. Assistive products and the Sustainable Development Goals (SDGs). Global Health. (2016) 12:79. 10.1186/s12992-016-0220-6 27899117 PMC5129191

[5] WHO EMRO. Strategic action framework to improve access to assistive technology in the Eastern Mediterranean Region. Cairo: World Health Organization Eastern Mediterranean Regional Office. (2020).

[6] Matter R Harniss M Oderud T Borg J Eide AH. Assistive technology in resource-limited environments: A scoping review. Disabil Rehabil Assist Technol. (2017) 12:105–14. 10.1080/17483107.2016.1188170 27443790

[7] Mishra S Laplante-Levesque A Barbareschi G Contepomi S Gowran R Kankipati P. Measuring met and unmet assistive technology needs at the national level: Comparing national database collection tools across eight case countries. In: Global Perspectives on Assistive Technology, Proceedings of the GReAT Consultation; 2019; Geneva, Switzerland. Geneva: World Health Organization (2019).

[8] Mishra S Laplante-Levesque A Barbareschi G De Witte L Abdi S Spann A. Assistive technology needs, access and coverage, and related barriers and facilitators in the WHO European region: a scoping review. Disabil Rehabil. (2022) 29:1–12. 10.1080/17483107.2022.2099021 35906719

[9] Zhang W Eide AH Pryor W Khasnabis C Borg J. Measuring self-reported access to assistive technology using the WHO Rapid Assistive Technology Assessment (rATA) questionnaire: Protocol for a multi-country study. Int J Environ Res Public Health. (2021) 18:13336. 10.3390/ijerph182413336 34948945 PMC8706997

[10] WHO. Measuring access to assistive technology in countries. Available online at: https://www.who.int/tools/ata-toolkit/rata (accessed April 2, 2024).

[11] WHO. Improving access to assistive technology: Resolution WHA 71.8. Geneva: World Health Organization (2018).32594831

[12] UNDP. Technical notes. Available online at: https://www.undp.org/sites/default/files/2021-22_HDR/hdr2021-22_technical_notes.pdf (accessed April 2, 2024).

[13] IBM Corp. IBM SPSS. Statistics for Windows. Version 280 Armonk, NY: IBM Corp. (2021).

[14] Baum F Musolino C Gesesew HA Popay J. New perspective on why women live longer than men: An exploration of power, gender, social determinants, and capitals. Int J Environ Res Public Health. (2021) 18:661. 10.3390/ijerph18020661 33466763 PMC7829786

[15] WHO. World report on disability. Geneva: World Health Organization (2010).

[16] Borg J Winberg M Eide AH Calvo I Khasnabis C Zhang W. On the relation between assistive technology system elements and access to assistive products based on 20 country surveys. Healthcare. (2023) 11:1313. 10.3390/healthcare11091313 37174855 PMC10178385

[17] Olarewaju T Healy A Chockalingam N. Barriers to accessing assistive technology in Africa. Assist Technol. (2023) 35:116–7. 10.1080/10400435.2021.1985011 34554890

[18] Eide AH Muller S Zhang W Khasnabisb C Antypasa K Blakstada M. “Barriers for accessing assistive products in low- and middle-income countries (LMICs),” In: D Archambault, G Kouroupetroglou (eds), *Assistive technology: Shaping a sustainable and inclusive world Amsterdam: IOS Press*. (2023). 10.3233/SHTI230634 37638928

[19] Senjam SS Mannan H. Assistive technology: The current perspective in India. Indian J Ophtalmol. (2023) 71:1804–9. 10.4103/IJO.IJO_2652_22 37203033 PMC10391423

[20] MacLachlan M. Access to assistive technology, systems thinking, and market shaping: A response to Durocher et al. Ethics Behav. (2019) 29:196–200. 10.1080/10508422.2018.1447382

[21] Olufadewa I Adesina M Ayorinde T. Global health in low-income and middle-income countries: a framework for action. Lancet Glob Health. (2021) 9:E899–900. 10.1016/S2214-109X(21)00143-1 34143987 PMC9237759

[22] Braveman P Gottlieb L. The social determinants of health: it's time to consider the causes of the causes. Public Health Rep. (2014) 129 Suppl 2:19–31. 10.1177/00333549141291S206 24385661 PMC3863696

[23] Krieger N. Theories for social epidemiology in the 21st century: an ecosocial perspective. Int J Epidemiol. (2001) 30:668–77. 10.1093/ije/30.4.668 11511581

[24] Layton N Brusco N Callaway L Henley L Wang RH. It is time for nationally equitable access to assistive technology and home modifications in Australia: an equity benchmarking study. Aust J Soc Issues. (2023) 59:244–63. 10.1002/ajs4.290

[25] Wang RH Wilson MG. It is time for a national strategy on equitable access to assistive technology in Canada. Healthc Manag Forum. (2022) 35:356–62. 10.1177/08404704221113742 35938298 PMC9615339

[26] Danemayer J Bloomberg M Mills A Holloway C Hussein S. Demographic, socioeconomic, and social barriers to use of mobility assistive products: a multistate analysis of the English Longitudinal Study of Ageing. Lancet Public Health. (2025) 10:e20–28. 10.1016/S2468-2667(24)00243-3 39675361 PMC11973444

[27] WHO. A conceptual framework for action on the social determinants of health. Geneva: World Health Organization. (2010).

[28] Danemayer J Boggs D Delgado Ramos V Smith E Kular A Bhot W. Estimating need and coverage for five priority assistive products: a systematic review of global population-based research. BMJ Glob Health. (2022) 7:e007662. 10.1136/bmjgh-2021-007662 35101862 PMC8804659

